# “Echoes of a dark past” is a history of maternal childhood maltreatment a perinatal risk factor for pregnancy and postpartum trauma experiences? A longitudinal study

**DOI:** 10.1186/s12884-023-05714-2

**Published:** 2023-05-29

**Authors:** Tracey Mackle, Lucía Colodro-Conde, Therese de Dassel, Anastasia Braun, Adele Pope, Elizabeth Bennett, Alka Kothari, George Bruxner, Sarah E. Medland, Sue Patterson

**Affiliations:** 1 Metro North Mental Health Service, Perinatal Wellbeing Team Brisbane, 10 Nellie Street, Nundah, QLD 4012 Australia; 2 QIMR Berghofer Medical Research Institute, Brisbane, QLD Australia; 3 University of Queensland, Brisbane, QLD Australia; 4 Redcliffe Hospital, Brisbane, Australia; 5 Metro North Mental Health Service, Brisbane, QLD Australia

**Keywords:** Adverse childhood experiences, Pregnancy, Postpartum, Perinatal outcomes, Post-traumatic stress disorder, Trauma

## Abstract

**Background:**

Although associations between maternal exposure to adverse childhood experiences (ACEs) and perinatal anxiety and depression are established, there is a paucity of information about the associations between ACEs and perinatal trauma and perinatal post-traumatic stress outcomes. For the purposes of this article, perinatal trauma is defined as a very frightening or distressing event that may result in psychological harm. The event must have been related to conception, pregnancy, birth, and up to 12 months postpartum.

**Methods:**

Women recruited at an antenatal appointment (*n* = 262) were invited to complete online surveys at two-time points; mid-pregnancy and eight weeks after the estimated date of delivery. The ACE Q 10-item self-reporting tool and a perinatal trauma screen related to the current and/or a previous perinatal period were completed. If the perinatal trauma screen was positive at either time point in the study, women were invited to complete a questionnaire examining symptoms of perinatal post-traumatic stress disorder and, if consenting, a clinical interview where the Post-traumatic Symptoms Scale was administered.

**Results:**

Sixty women (22.9%) reported four or more ACEs. These women were almost four times more likely to endorse perinatal trauma, when compared with those who either did not report ACEs (OR = 3.6, CI 95% 1.74 – 7.36, *p* < 0.001) or had less than four ACEs (OR = 3.9, CI 95% 2.037.55, *p* < 0.001). A 6–sevenfold increase in perinatal trauma was seen amongst women who reported having at least one ACE related to abuse (OR = 6.23, CI 95% 3.32–11.63, *p* < 0.001) or neglect (OR = 6.94, CI 95% 2.95–16.33, *p* < 0.001). The severity of perinatal-PTSD symptoms for those with perinatal trauma in pregnancy was significantly higher in those women exposed to at least one ACE related to abuse.

**Conclusions:**

Awareness of maternal exposure to childhood adversity/maltreatment is critical to providing trauma-informed approaches in the perinatal setting. Our study suggests that routine screening for ACEs in pregnancy adds clinical value. This adds to previous research confirming the relationship between ACEs and mental health complexities and suggests that ACEs influence perinatal mental health outcomes.

**Supplementary Information:**

The online version contains supplementary material available at 10.1186/s12884-023-05714-2.

## Background

Early detection of risk factors negatively affecting maternal mental health in the perinatal period is widely accepted as best practice [1–3]. Typically, psychosocial screening encompasses exploration of current depression and anxiety symptoms, mental health history and factors known to influence perinatal adjustment including intimate partner violence, financial difficulties, and lack of emotional, practical, and social supports. Maternal exposure to adverse childhood experiences (ACEs), including abuse, neglect and family dysfunction, is not routinely screened for during perinatal care in Australia [4]. This is despite mounting evidence that ACEs before the age of 18 years have profound adverse effects on health outcomes in general populations [5] and substantially increase risk of mental health problems and suicide attempts across the lifespan [5–7].

The perinatal period is recognised as a time of increased vulnerability for emergent or relapsing mental health conditions [8, 9]. Causation is inherently complex, but increased risk is associated with substantial neuro-endocrinological fluctuations [10], rapid physiological changes, and role and relationship transitions that may negatively impact a woman’s sense of control and resilience [10–12]. Poor maternal mental health during pregnancy increases risk of obstetric complications, including gestational diabetes, preterm birth, intra-uterine growth restriction, and low neonatal birth weight [10, 13–15]. In the postpartum, poor maternal mental health increases risk of breastfeeding difficulties, maternal and newborn stress [16], poor mother-infant bond, disrupted and disorganised attachment in infants [17–19] as well as maternal mortality [20].

Pregnancy and childbirth complications may be experienced as traumatic and have the potential to harm maternal psychological well-being resulting in symptoms of post-traumatic stress disorder. Such events include: difficulty conceiving naturally, severe nausea and vomiting during pregnancy or hyperemesis gravidarum [21–23], diagnosis of foetal anomaly [24], intra-uterine growth restriction [25] and maternal medical complications [13]. Obstetric complications include premature birth, miscarriage, intrauterine foetal demise, stillbirth and neonatal death [26], severe maternal morbidity and pain [27]. Women may also experience trauma following negative or invalidating interactions with healthcare providers, unmet plans and expectations for labour, and the need for unplanned medical interventions [28]. For the purposes of our study, perinatal trauma is defined when women endorse a very frightening or distressing event that may result in psychological harm [29]. The event must have been related to conception, pregnancy, birth, or 12 months postpartum.

Post-Traumatic Stress Disorder (PTSD) refers to a cluster of symptoms that develop after exposure to a traumatic event involving actual or threatened death, serious injury or sexual violence. Research into PTSD during the perinatal period has focused predominantly on the development of the disorder following a difficult birth during which women believe they or their baby might die or be seriously hurt [30] but there is paucity of studies exploring other phases of the perinatal period. Limited attention has been focussed on maternal ACEs and whether this is an added risk factor for women experiencing perinatal trauma and developing perinatal PTSD symptoms, despite research showing elevated risk for developing PTSD after ACEs [31]. This knowledge gap hinders both design and implementation of best practices for perinatal care and maternal and infant outcomes.

### Aims

Aiming to inform practice improvement, we set out to a) identify the prevalence of ACEs in a cohort of pregnant Australian women, b) assess the association between ACEs and the incidence of perinatal trauma and c) assess the association between ACEs and PTSD symptoms for those women who had perinatal trauma.

## Methods

### Methodological approach

**Fig. 1:**
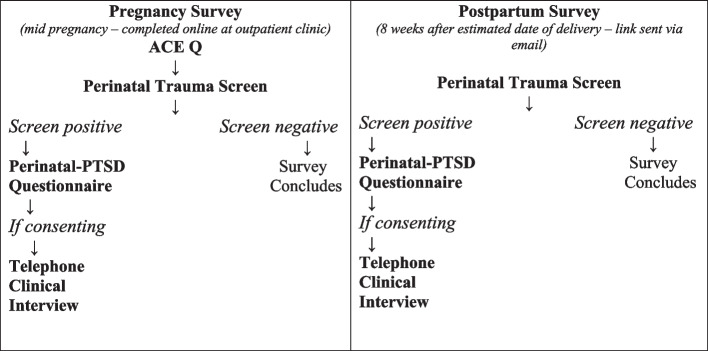
Longitudinal study time points and completion of measures by participants

This was a longitudinal study including both self and clinician administered questionnaires. Participants were invited to complete online surveys at two time points: during pregnancy (pregnancy survey) and eight weeks following the estimated date of delivery (postpartum survey). The pregnancy survey included questions on demographics and the ACE Q. In both surveys, women were screened for perinatal trauma occurring during conception, pregnancy, birth or the 12 months postpartum. If the perinatal trauma screen was positive, participants were invited to complete an online questionnaire examining perinatal-PTSD symptoms occurring over the last month, and if consenting, a telephone clinical interview administering the Post-traumatic Symptom Scale (PSS-I-5) [32] was completed. See Fig. 1 for study time points and completion of measures.

The goal of the researchers was to inform and extend clinical practice in the detection and support of women experiencing trauma related to perinatal events and/or perinatal-PTSD. Therefore, the research team approached the study from a trauma informed lens. This approach is grounded in acknowledgement that trauma is subjective, prevalent and may cause psychological harm which impact a person’s physical, emotional, and mental health and help-seeking behaviours [31].

### Participant recruitment

Participants were recruited from the maternity outpatient clinic of an outer metropolitan public hospital in Queensland, Australia, between November 2019 and March 2020. Participants were eligible if they were pregnant, attended the clinic for an antenatal appointment during the recruitment period, were 18 years or older, and sufficiently fluent in English to complete the consent process and surveys. Potential participants were approached in the waiting room by a researcher not involved in the provision of healthcare to the women and provided with verbal information about the study. Those who expressed interest in participation were given a portable electronic device to read a participant information sheet and complete a consent form before proceeding to the online survey. Information sheets were provided at all data collection points of the study, with written consent obtained at each contact. All participants were provided with details of how to access further mental health support/counselling after each contact and were advised they could withdraw consent at any time.

### Measures

#### ACE Q

The ACE Q comprises of ten yes/no questions around abuse (physical, sexual and emotional), neglect (physical and emotional) and dysfunctional family environment (parental mental illness, parental divorce/separation, parental substance use, maternal violence and incarceration of a family member) prior to the age of 18 years [33]. The ACE score is calculated from 0 being no exposure to 10 exposures, with the overall score used in analyses. Over the past two decades, the ACE Q has demonstrated strong predictive value, good reliability, and internal consistency. However, the ACE Q does not measure the degree, duration or severity of ACEs [33].

### Perinatal trauma screen

Women were asked to endorse if at any time during the perinatal period they felt “afraid/distressed that something bad was happening or going to happen, and/or that their or their baby’s life was in danger”. If a positive response was given, they were asked to identify whether this was during the current or a previous perinatal period or both. To further define perinatal trauma they were asked to select at least one event from the following list: afraid they might die, afraid they were in danger, a health care professional expressed concerns with their health or they experienced any injury related to the perinatal period; and/or they were afraid their baby might die, afraid the baby might be in danger, and/or a health care professional expressed concerns about the baby’s health, growth or development. This list of perinatal trauma events was guided by the consultation process described in the next section and aimed to address the DSM-5 PTSD Criteria A: Exposure to actual or threatened death, serious injury or sexual violence. We did not specify events like “birth trauma” or ask direct questions about whether the women felt traumatised (See Additional file Section One for the questions included in the perinatal trauma screen).

### Perinatal-PTSD questionnaire

This questionnaire was developed by the study team in consultation with 21 consumer representatives and 57 perinatal health care professionals who completed a modified Delphi method survey. In addition to the perinatal trauma screen described above, a further 96 questions developed as a first step by the research-clinical team were voted by the stakeholders as useful or not useful in addressing each of the additional DSM-5 PTSD criteria; associated with and beginning after the Criteria A event(s); this included Criteria B: intrusion symptoms, C: avoidance symptoms, D: negative alternations in cognitions and mood symptoms, and E: marked alteration in arousal and reactivity symptoms [34].

The two questions voted most useful for each of the criteria were discussed in a follow-up consultation forum. We also identified if either of these questions were preferred by the consumer representative group vote before the 20 questions were finalised. Five additional questions related to perinatal trauma symptoms were included, although in this study we only scored the questions addressing PTSD criteria. Participants could indicate, in thinking about the event specified in the perinatal trauma screen, how often they experienced each of the symptoms over the last month (not at all, once or twice, several days, more than half the days, nearly every day). A score indicating severity of the current impact of the perinatal trauma was calculated by computing a weighted sum of the frequency of the symptoms, with scores ranging from 0 to 80, where higher scores indicate higher severity. The internal consistency was α = 0.97.

This is not a validated questionnaire but part of a project aiming to develop a screening tool for perinatal-PTSD (see Additional file Section One for the perinatal-PTSD questionnaire used in this study).

### Post traumatic symptom scale interview

The validated PSS-I-5 [32] clinical interview was administered by telephone to further assess the impact of the perinatal trauma, current symptom severity and for a diagnosis of PTSD. PTSD severity is computed by totalling the 20 PSS-I-5 symptoms ratings (scores range from 0–80). Participants with scores of 23 or more were defined as PTSD cases (cut off for probable PTSD diagnosis by the authors of the scale). A PTSD diagnosis was given when participants had a score of 1 or greater in avoidance and intrusion symptoms and a score of 1 or greater in two cognition/mood symptoms and two arousal/reactivity symptoms, in addition to symptom duration of more than one month with clinically significant distress or impairment (score of 2 or more)*.* Both the pregnancy and postpartum interviews were completed by a psychologist who was not involved in the provision of healthcare to the women and was blinded to their results in the perinatal-PTSD questionnaire, other than a positive screen to perinatal trauma.

### Statistical analysis

Statistical analyses were performed in SPSS v 23 [35]. A *p*-value of < 0.01 was used as the criterion for statistical significance. Descriptive analyses were conducted to summarise the women’s demographic characteristics, the ACE Q and perinatal trauma event(s) endorsed by the women using standard methods.

**Fig. 2:**
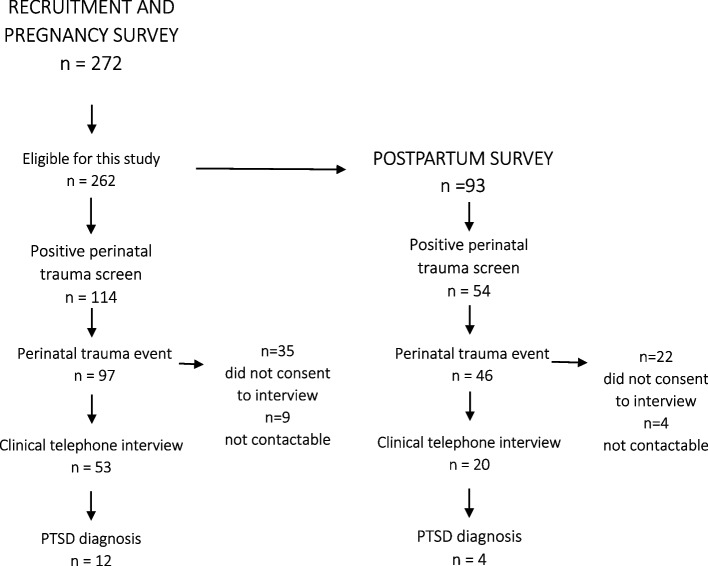
Flowchart of the participants involved in each phase of the study and main outcomes. Only participants endorsing perinatal trauma went on to complete the perinatal-PTSD questionnaire and clinical interview. See Table 2

To explore the relationship between ACE and perinatal trauma, we restricted the analyses to those participants with a positive screen to perinatal trauma and reporting at least one perinatal event from the list (see Fig. 2). Unadjusted odds ratios (Pearson Chi-Square or Fisher’s Exact Probability statistic tests of significance) were calculated for perinatal trauma for each of the ACEs versus no ACEs or any other ACEs, for reporting at least one ACE related to abuse, to neglect and to household dysfunction versus no ACEs of that kind and increases in total number of ACEs versus no ACEs.

The total ACE score and number of neglect, abuse, and household dysfunction related ACEs were compared between women with perinatal trauma in the pregnancy and/or in the postpartum survey and those with no perinatal trauma at any point of the study using Student’s t-Tests for independent samples. Multiple logistic regressions with perinatal trauma as outcomes were conducted, where potential socio-demographic confounders (age, level of education, SES of place of residence, parity, being in a relationship, being in paid employment at time of recruitment) were included as predictors together with ACE variables, with results reported as Additional material.

To explore the relationship between ACEs and PTSD symptoms in women with perinatal trauma, we limited our analyses to the pregnancy responses. We first obtained the correlation of the severity score provided by the two measures and the accuracy of the severity score of the perinatal-PTSD questionnaire in relation to a probable diagnosis of PTSD from the PSS-I-5 using logistic regression. The severity score from the perinatal-PTSD questionnaire presented a moderate correlation with that of the PSS-I-5 (*r* = 0.53, *p* < 0.001) and correctly classified 79.2% with and without a probable diagnosis or a diagnosis of PTSD according to the results of the clinical interview (*n* = 53). Therefore, to maximise the sample size we used the severity score from the pregnancy survey perinatal-PTSD questionnaire to study the relationship between ACEs and perinatal post-traumatic stress (*n* = 96). The mean of PTSD severity in the perinatal-PTSD questionnaire was compared to women reporting at least one ACE related to abuse, to neglect and to household dysfunction using Student’s t-Tests for independent samples. Multiple linear regression models with PTSD severity as outcome were conducted, where potential social confounders (age, level of education, social economic status (SES) of place of residence, parity, being in a relationship and being in paid employment at the time of recruitment) were included as predictors together with ACE variables, with results reported as Additional material.

## Results

### Demographics

Three hundred and eighty approaches were made to potential participants during November 2019 and March 2020. Twenty-four women were not eligible (less than 18.years old or not fluent in English), 52 women approached had already completed the survey and 34 declined to participate. A total of 272 women consented to participate. This study was restricted to 262 participant’s completing basic demographics and the ACE Q self-report in the pregnancy survey. Of these women, 93 also participated in the postpartum survey. See Fig. 2 for study flowchart.

The mean age of participants was 28.4 years (SD = 5.2, range 18), predominantly of Caucasian background (68.7%), with only 8.8% reporting they had not completed senior secondary education. Most (64.9%) were in paid employment and in a relationship (83.9%). We linked the postcode of residence to the Index of Relative Socio-Economic Advantage and Disadvantage to obtain a measure of socioeconomic well-being in a continuum, from the most disadvantaged areas (low values) to the most advantaged areas (high values). This index estimates access to material and social resources and a person’s ability to participate in society in their neighbourhoods. We used deciles of this index, which allowed comparison within postal areas in the same state [36]. Regarding the SES of the place where women resided, the median was 7 (mean = 6.4, SD = 2.8, range = 1–10), indicating that participants tended to live in areas with greater advantage in general [36]. The mean pregnancy week was 28.4 (SD = 5.2, range = 18–41) and 39.7% of the participants had no children at the time of recruitment. See Table 1 for demographic details at recruitment.

**Table 1:** Demographics at recruitment (*n* = 262)

	**Frequency (%)**
**Identifies as**	
Aboriginal or Torres Strait Islander	13 (5%)
Caucasian/White	180 (68.7%)
Polynesian	16 (6.1%)
Asian	11 (4.2%)
Indian	6 (2.3%)
Other	29 (11.1%)
Missing	7 (2.7%)
**Level of education**	
Junior secondary school (years 8–10)	23 (8.8%)
Senior secondary school (years 11–12)	79 (30.2%)
Certificate or diploma	85 (32.4%)
Degree	60 (22.9%)
Post Graduate Diploma, Master’**s** degree	15 (5.7%)
**Main occupational activity**	
Student attending university or other tertiary education	13 (5%)
Full-time or part-time work	170 (64.9%)
Apprenticeship/ Traineeship	3 (1.1%)
Unemployed/ looking for work	12 (4.6%)
Home duties	50 (19.1%)
Have a job, but not at work due to illness, vacation, etc	8 (3.1%)
Not working and currently receiving sickness allowance or disability support pension	3 (1.1%)
Other	3 (1.1%)
**Relationship status**	
Single	31 (11.8%)
Separated/Divorced	8 (3.1%)
Widowed	1 (0.4%)
De facto or Married (male partner)	212 (80.9%)
De facto or Married (female partner)	8 (3.0%)
Missing	2 (0.8%)
**Number of children**	
0	104 (39.7%)
1	95 (36.3%)
2	41 (15.6%)
3	13 (5%)
4	7 (2.7%)
5	1 (0.4%)
6	1 (0.4%)

### Adverse childhood experiences

**Fig. 3:**
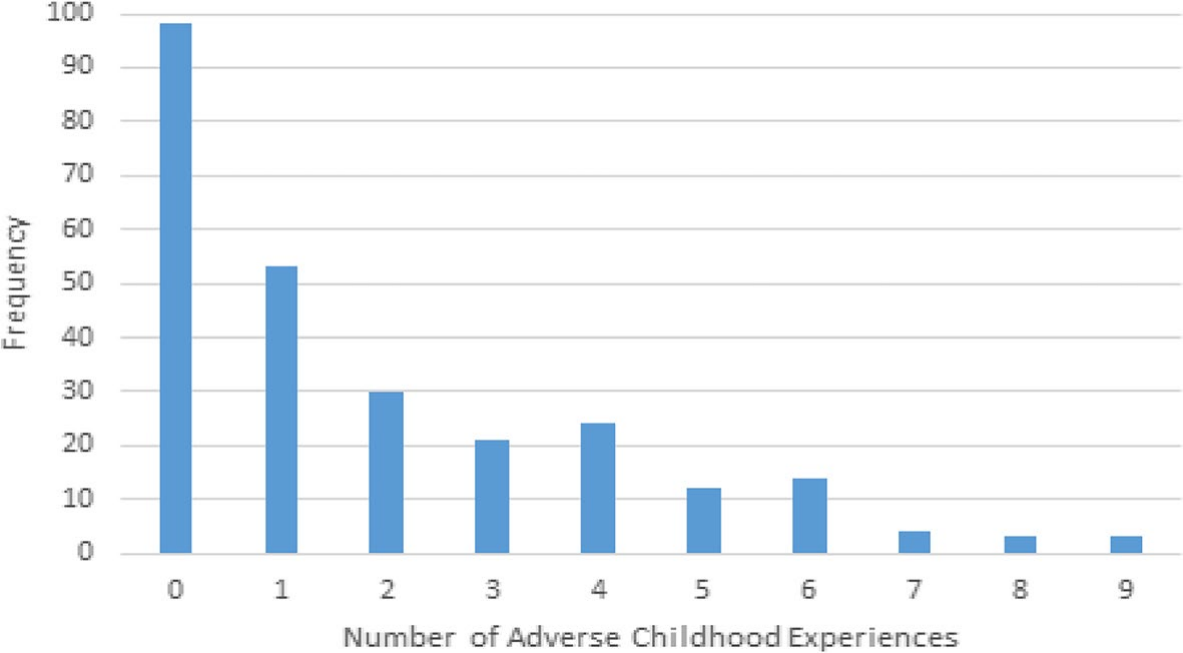
Total ACEs reported: 0 being no ACE and 9 being the highest cumulative ACEs result recorded by a participant in the pregnancy survey (*n* = 262)

Exposure to at least one ACE was reported by 62.6% women, and the mean number of ACEs was 1.89 (SD = 2.2, range = 1–9). The least prevalent ACE reported was physical neglect at 3.1% with parental separation or divorce being the most prevalent at 50.8%. Parental mental illness (38%) and substance use (23%) were common. Nearly a quarter (22.9%) of the women reported four or more ACEs. The highest cumulative ACEs score was 9, reported by three individuals. See Table 2 (first panel) and Fig. 3 for more details of ACEs.

**Table 2:** Endorsement of ACEs (*n* = 262) in first panel and association with positive perinatal trauma screen at any time in the study (*n* = 122) in the second panel

ACEs	Frequency (%)	OR (CI 95%, *p*-value)
**Abuse**		
Emotional	62 (23.7%)	5.78 (2.88—11.65, *p* < 0.001)
Physical	38 (14.5%)	4.28 (1.87—9.84, *p* < 0.001)
Sexual	33 (12.6%)	4.49 (1.87–10.79, *p* < 0.001)
**Neglect**		
Emotional	44 (16.8%)	7.84 (3.16–19.48, *p* < 0.001)
Physical	8 (3.1%)	7.49 (0.91–61.84, *p* = 0.035)*
**Household Dysfunction**		
Parental divorce/separation	133 (50.8%)	0.86 (0.52—1.42, *p* = 0.57)
Witness to Maternal violence	27 (10.3%)	2.21 (0.95–5.14, *p* = 0.06)
Parental substance use	61 (23.3%)	3.156 (1.67–5.96, *p* < 0.001)
Parental mental illness	75 (38.6%)	1.69 (0.97, 2.94, *p* = 0.63)
Incarceration of a family member	14 (5.3%)	0.86 (0.28–2.63, *p* = 0.79)

Note participants could report more than one ACE. ^*^Fisher’s test of significance; the rest are Pearson's tests

With consideration to the acceptability of ACE screening, women in our study were able to proceed through the ten-item questionnaire without compulsory fields, meaning they could skip any question if they did not wish to answer it. Of the 262 completions, there were one or two responses missing for 9 individuals. These data appeared to be missing at random. In this instance, we adopted a conservative approach and assigned a no response rather than removing the women from the data.

### Perinatal trauma

In the pregnancy survey, 97 (39.6% of the total sample) had a perinatal trauma (a total of 17 women had a positive screen to perinatal trauma but did not specify the type of perinatal trauma event and were excluded from the analyses). Among those, 45 (46.4%) reported at least one perinatal trauma related to themselves and at least one related to their baby. Of those completing the postpartum survey, 46 (54.1%) had perinatal trauma and among these, 23 (50%) reported perinatal trauma that had affected themselves and their baby. In summary, a total of 122 women (53.1% of the total sample) had perinatal trauma, either only in the pregnancy survey (*n* = 76), only in the postpartum survey (*n* = 25) or in both (*n* = 21). Please see Table 3 for the trauma characteristics in participants screening positive to perinatal trauma.

**Table 3:** Trauma characteristics in participants screening positive to perinatal trauma in the surveys (frequency and percentage)

	**Pregnancy survey** **(*n* = 97)**	**Postpartum survey** **(*n* = 46**
**Perinatal phase when perinatal trauma endorsed**		
Conception	12 (12.4%)	6 (13.0%)
Pregnancy	74 (76.3%)	25 (54.3%)
Birth	29 (29.9%)	24 (52.2%)
Postpartum	21 (21.6%)	21 (45.6%)
**Perinatal period when perinatal trauma endorsed**		
Current	40 (41.2%)	24 (52.2%)
Past	27 (27.8%)	3 (6.5%)
Current and Past	29 (29.9%)	15 (32.6%)
Missing	1 (1%)	4 (8.7%)
**Perinatal Trauma event**		
1. Afraid of dying (self)	20 (20.8%)	11 (23.9%)
2. Afraid in danger (self)	26 (27.4%)	12 (26.1%)
3. Concerns from professionals about health (self)	29 (30.5%)	10 (21.7%)
4. Injuries related to perinatal period (self)	10 (10.5%)	9 (19.6%)
5. Afraid of baby dying	77 (79.4%)	33 (71.7%)
6. Afraid baby in danger	73 (75.3%)	33 (71.7%)
7. Concerns from professionals about baby health, growth/development	24 (25%)	11 (23.9%)

### Relationship between adverse childhood experiences and perinatal trauma

**Fig. 4:**
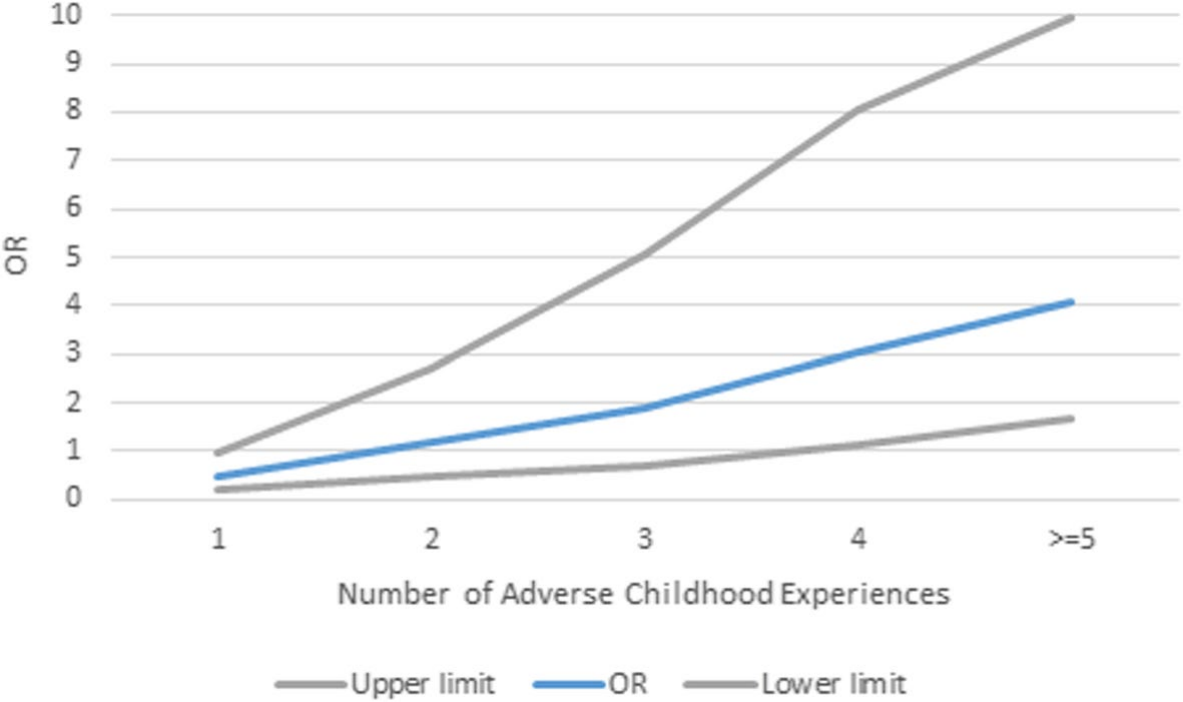
Odds ratio (OR) and 95% confidence intervals of positive screen for perinatal trauma for individual ACEs

We analysed the relationship between ACEs and perinatal trauma at any point in the study (*n* = 122 exposed and *n* = 123 not exposed). A positive score on most ACEs, especially those related to abuse and neglect, was associated with perinatal trauma (see Table 2, second panel). Exposure to at least one ACE related to abuse (OR = 6.23, CI 95% 3.32–11.63, *p* < 0.001) or neglect (OR = 6.94, CI 95% 2.95–16.33, *p* < 0.001) represented over a six-fold increase in women endorsing perinatal trauma; however, the exposure to at least one household dysfunction ACEs did not increase the risk (OR = 1.17, CI 95% = 0.70–1.97, *p* = 0.55). The likelihood of perinatal trauma increased with the number of ACEs reported (see Fig. 4 for the odds ratio and confidence intervals). The total ACE score was higher in women with perinatal trauma (t(207.69) = -4.53, *p* < 0.001). The number of ACEs related to abuse (t(203.20) = -5.90, *p* < 0.001) and neglect (t(159.74) = -5.15, *p* < 0.001) was also higher in women with perinatal trauma. The number of ACEs related to household dysfunction did not differ in women with or without perinatal trauma (t(234.78) = -2.03, *p* = 0.43). After controlling for socio-demographic variables in multiple regression models the total number of ACEs (OR = 1.41, CI 95% 1.22, 1.63, *p* < 0.001), ACEs related to abuse (OR = 3.1, CI 95% 2.04, 4.72 *p* < 0.001) and neglect (OR = 7.75, CI 95% 3.24, 18.52, *p* < 0.001) predicted perinatal trauma (see Additional File—Sect. 2, Tables 1–4).

Moreover, women with four or more ACEs compared with women who did not report ACEs (OR = 3.6, CI 95% 1.74 – 7.36, *p* < 0.001) or had less than four ACEs (OR = 3.9, CI 95% 2.03, 7.55, *p* < 0.001) had an almost four-fold increased probability of reporting perinatal trauma. These results remained consistent after controlling for socio-demographic variables (see Additional File Sect. 2 – Tables 5–6).

### Relationship between adverse childhood experiences and perinatal post-traumatic stress symptoms

The following analyses were restricted to the responses provided in the pregnancy phase of the study. The severity score of perinatal-PTSD symptoms from the questionnaire had a mean score = 14.9, SD = 15.1, range = 0–57, n = 96) and presented a moderate correlation (*r* = 0.53, *p* < 0.001, *n* = 53) with the total score of the PSS-I-5 used in the clinical interview (mean = 14.6, SD = 14.1, range = 0–53). See Table 4 for the correlations between ACEs and perinatal-PTSD severity according to the perinatal PTSD questionnaire.

**Table 4:**
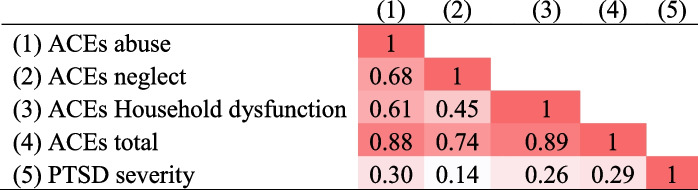
Correlations between ACEs and Perinatal-PTSD severity

Darker cell colours indicate higher correlations. All correlations were significant (*p* < 0.01) except perinatal-PTSD severity and neglect

The severity of perinatal-PTSD symptoms for those with perinatal trauma in pregnancy was significantly higher in those women exposed to at least one ACE related to abuse (t(91.57) = -3.40, *p* < 0.001) but not for those reporting at least one ACE related to neglect (t(94) = 1.60, *p* = 0.11) or household dysfunction (t(94) = 1.69, *p* = 0.09). These results remained consistent after controlling for socio-demographic variables in multiple regression models (see Additional File Sect. 2 – tables 7–9).

## Discussion

Our study showed a high prevalence of participants reporting four or more ACEs. Of the 262 women, 22.9% (*n* = 60) reported four or more ACEs and 9.2% (*n* = 24) reported scores of six or more indicating a significant burden of health risk [37]. This finding is comparable to other national and international ACE studies [37–40]. ACEs were related to perinatal trauma and severity of PTSD symptoms. Identifying women at risk during the perinatal period is important because ACEs have been demonstrated to increase susceptibility to depression and anxiety [11, 39], suicide [5–7] and post-traumatic symptoms [25, 39] and as our study shows, perinatal trauma and perinatal-PTSD symptoms. ACEs and co-morbid maternal mental health conditions may have a detrimental impact on resilience and coping with the stressful experiences that are inherent to pregnancy, giving birth and transitioning to parenting [12].

Evidence suggests women who experience childhood abuse are at higher risk for being triggered by the events of pregnancy and or birth [14, 21, 41]. Our study findings provided further support for this understanding, showing a positive association between four or more ACEs and perinatal trauma, with a four-fold increase of such women reporting perinatal trauma compared to women with less than four or no ACEs. A positive screen for perinatal trauma was highly prevalent in our pregnancy sample (*n* = 114) and 37% of the total study participants endorsed at least one perinatal trauma event.

During pregnancy, *n* = 14 women with perinatal trauma had a probable diagnosis of perinatal-PTSD and *n* = 12 met the DSM-5 criteria for diagnosis of PTSD. The most frequently endorsed perinatal trauma event during pregnancy was a fear that their unborn child was in danger (75%) or was going to die (79%). This contrasts with the much lower rate of women endorsing perinatal trauma related to a health care professional(s) advising of actual concerns regarding maternal health issues during pregnancy (30%) or issues with foetal growth and development (25%). These contrasting figures remained consistent in the postpartum survey. In other perinatal studies, women with four or more ACEs were more likely to present with pregnancy problems [10], have more unplanned or frequent contact with the health care system [4] and experience perinatal related anxiety [39]. Our findings are important, as these fears left undetected and unaddressed can have detrimental effects on the mother and infant’s physical and mental health outcomes in both the pregnancy and postpartum. Moreover, our study highlights a missed opportunity to offer trauma orientated care and additional emotional support for women who are triggered by past childhood experiences or memories.

Association between ACEs, reproductive health issues and adverse pregnancy outcomes has been widely established [8, 14, 16, 25, 42–44]. Yet uptake of ACE screening into clinical practice and acknowledgement of its contribution to predicting cumulative perinatal health risk has not occurred in the Australian maternity context [4]. Our study contributes to the evidence base that ACE screening should be considered in clinical practice in the perinatal setting, given the prevalence in the population and the potential to prompt early intervention measures. Historically, concerns have been expressed that ACE screening is invasive and may cause patient discomfort [5]. However, international literature does not reflect this finding, with many study participants reporting acceptability of being screened using ACE self-reports [45] particularly during pregnancy and when benefit was identified for their infant [44].

High maternal ACEs strongly correspond with negative impacts on the mother and infant bond [30, 46] contributing to breast feeding difficulties, and maladaptive maternal coping [4, 11, 16, 47]. Thus, a greater focus is needed on caregivers’ childhood experiences to better support infants’ development. In a recent review, infants whose mothers had four or more ACEs were found to have a fivefold increase in physical and emotional health difficulties at 18 months [45]. Extrapolating those findings to the current cohort, early identification of women experiencing perinatal trauma and subsequent targeted intervention to support emotional well-being may have important long-term benefits to a sizeable proportion of the study population (almost 23%) and their infants.

To extend the benefit of trauma focussed care, future research could explore the impact of ACEs in the conception phase, as perinatal trauma was endorsed by over 10% of participants during this time frame.

### Strengths and limitations

Firstly, a strength of our study was the type of research sample, which included women attending a mid-pregnancy antenatal appointment in an outer metropolitan setting, inclusive of both nulliparous and parous women. Secondly, the study was positively received with a high willingness of participation. There are some limitations to our study. Majority of the participants were of Caucasian background, middle-class and therefore may not be representative of other settings with greater economic, ethnic, and cultural diversity. Despite this, there was high prevalence of ACEs in this sample comparable with other national and international ACEs research. Another limitation of the study is that the ACE Q is a retrospective self-report and may be subject to recall bias, however in other studies this has not been the trend and recall of childhood abuse appears consistent over time [5, 48].

Whilst the ACE Q captures the most common ACEs, it does not capture all types of childhood trauma that can impact on lifelong risk factors such as loss/death of a loved one, serious accidents, severe illness, bullying/peer rejection, extreme poverty and impacts of disasters; war, floods, pandemics [49]. The study only controlled for socio-demographic variables and did not collect others (e.g. current obstetric risks) which may influence positive screening outcomes. We did not collect mitigating factors that promote an individual’s resilience and coping in adulthood [12, 44] which may reduce the negative impact or risk associated with cumulative ACEs and experience of perinatal trauma/ perinatal-PTSD. Seventeen women were excluded from the analyses as they had a positive screen to perinatal trauma but did not choose an event(s) from the predefined list. Having fixed fields in the screen may have maximised the sample size by 14%, however it is possible that this was not completed as the predefined event(s) did not fit with the individual’s perinatal experience. In the study design we hypothesised there would be a risk for women to drop out in the postpartum period, acknowledging new mothers are time poor, often have their mobile devices on silent and answering a survey may not be a priority. This hypothesis was realised, and with only a 35% response rate our postpartum sample size was reduced.

## Conclusion

ACE screening provides valuable data to further inform maternal mental health risk and enable appropriate care planning during antenatal and postpartum care. It aligns with a trauma-informed approach of exploring “what happened to you” versus the perception of “what is wrong with you” [50]. Exploration of maternal childhood adversity/maltreatment identified through ACE screening allows for discussion and reflection of a woman’s experience of ACEs, the threat or survival responses they have developed [47, 50], potential impacts on their mental health, parenting journey and mother infant bond. Greater awareness of the widespread impact of ACEs on both emotional and physical health is imperative for all clinicians involved in obstetric care given the strong association with perinatal morbidity. With the unprecedented access health professionals have to individuals during the perinatal period comparative to other life stages, implementation of ACE screening in pregnancy would appear to offer high clinical value despite reported impediments to implementing additional screening. Ability to identify individuals who are predisposed to higher multifactorial health risks secondary to ACEs and provision of additional emotional supports during antenatal care could reduce the experience of perinatal trauma/perinatal-PTSD, other negative mental health outcomes, long term consequences and intergenerational ACEs exposure for the infant.

## Supplementary Information

**Additional file 1.**

## Data Availability

The datasets generated and/or analysed during the study are available from the corresponding author on reasonable request.

## Abbreviations

ACEs: Adverse childhood experiences
DSM-5: Diagnostic Statistical Manual of Mental Disorders Fifth Edition
PTSD: Post-traumatic stress disorder
Perinatal-PTSD: Perinatal post-traumatic stress disorder
PSS-I-5: Post-traumatic Symptom Scale Interview for DSM-5
SES: Social Economic Status
