# Supplementary material for: “Echoes of a dark past” is a history of maternal childhood maltreatment a perinatal risk factor for pregnancy and postpartum trauma experiences? A longitudinal study
Source: BMC Pregnancy Childbirth. 2023 May 29;23:397. doi: 10.1186/s12884-023-05714-2 (PMC10226204; doi:10.1186/s12884-023-05714-2)
Supplement: Supplementary file 1 — Additional file 1. [file 12884_2023_5714_MOESM1_ESM.docx]

**Additional Files**

***Section One***

***Perinatal Trauma Screen***

This questionnaire asks about your experience/s while trying to get pregnant, during pregnancy, birth, and the first year afterwards. This is defined as **the perinatal period**.

The term *baby* is used broadly in this questionnaire and includes your unborn child.

We are interested in any experience during your perinatal period/s where you have felt: afraid/distressed something bad was happening or going to happen, and/or that your or your baby’s life was in danger? (*you can tick more than one item)*

- Yes, trying to conceive
- Yes, during pregnancy
- Yes, during birth
- Yes, after the birth
- No *(survey concludes and Thank-you message was generated)*

Was this during the current perinatal period?

Yes/No

Did this occur in a past perinatal period?

Yes/No

Were you afraid that you might die?

Yes/No

Were you afraid that you were in danger?

Yes/No

Were you afraid that the baby might die?

Yes/No

Were you afraid the baby might be in danger?

Yes/No

Did a professional express concern about the baby's growth or development?

Yes/No

Did a professional express concern with your health?

Yes/No

Did you experience any injury related to the perinatal period?

Yes/No

Have you ever spoken to a health care professional about this experience?

Yes/No

(*if previous answer is no*)

Would you like to speak to a health care professional about this experience?

Yes/No

***Perinatal-PTSD Questionnaire***

**Please read each item carefully.** In thinking about what happened to you or your baby as described above; please tell us how often you experienced each of the following during **THE LAST MONTH.**

Options of response are:

**Not at all Once or twice Several days More than half the days Nearly every day**

1. Having upsetting thoughts or images come into your head when you do not want them
2. Waking up feeling upset after a bad dream
3. Suddenly feeling or acting as if what happened to you and/or your baby was happening again (i.e. as if you were re-experiencing by seeing, hearing, smelling, or having physical sensations)
4. Feeling distressed when something reminds you of what happened to you and/or your baby (i.e. smells, sounds, conversations, seeing pregnant women, seeing babies, thoughts, feelings, body sensations, pain)
5. Experiencing physical reactions when something reminds you of what happened to you and/or your baby (i.e. sweating, increased heart rate, heart pounding, short of breath, nausea, dizziness, lightheaded, tense)
6. Avoiding or trying to avoid thoughts, feelings, physical sensations, that remind you of what happened to you and/or your baby
7. Avoiding or trying to avoid events or circumstances that remind you of what happened to you and/or your baby (i.e. medical appointments, people, places, newborns, health care professionals)
8. Having trouble remembering important parts of the experience or feeling there are blanks in memory
9. Having strong negative beliefs about yourself, other people, or the world (i.e. there is something seriously wrong with me or my baby, something bad is going to happen, no one can be trusted)
10. Feeling guilty or unable to stop blaming yourself or others for what happened or any problems that have followed to you and/or your baby
11. Having strong negative feelings such as fear, grief, resent, disappointment, anger, guilt, or shame, or feeling broken or damaged
12. Feeling you have lost interest or are unable to do things you enjoy or usually do (i.e. tasks around the house, caring for newborn, socialising with friends, shopping)
13. Feeling numb, alone even when supported, or disconnected from yourself or your body, people, activities, or your surroundings
14. Not being able to feel joy, delight, happiness, excitement, or loving feelings (i.e. towards baby, partner, family members, friends)
15. Feeling irritated or cranky, or having sudden episodes of anger with no obvious or clear trigger
16. Being more reckless and not caring that what you are doing could cause you harm (i.e drinking, taking drugs, driving too fast)
17. *Thinking of hurting yourself or somebody else

(if other than ‘Not at all’): *If you are feeling this way, discussing your thoughts and feelings with a counsellor may help. Lifeline has telephone counsellors, available 24 hours a day. 13 11 14*

1. Being ‘super alert’, ‘on guard’, or constantly on the lookout for something dangerous or going wrong
2. Feeling jumpy or easily startled when you hear an unexpected noise (i.e. baby noises, noises outside, appliances beeping)
3. Having problems paying attention or getting things finished
4. Having troubles falling asleep or not sleeping well even when feeling exhausted.

(if other than ‘Not at all’): *Is this related to physical discomfort, the pregnancy or the baby? Yes or No*

1. *Feeling that you are zoning out, losing time, or not being present
2. *Not trusting that health care professionals are providing you or your baby adequate care
3. *Feeling powerless, out of control, or that your needs were not met
4. * Feeling as if your future hopes or plans are no longer possible

The five questions denoted with a * were not used in the severity score for perinatal-PTSD. This was a digital survey so the italic items are generated when anything other than “not all” was selected.

**Section Two**

**Table 1.** *Regression coefficients for predicting perinatal trauma, including the total ACE score.*

|  | B | SE B | Wald χ2 | p-value | OR | 95% CI OR |
| --- | --- | --- | --- | --- | --- | --- |
| Constant | -1.931 | 1.016 | 3.613 | 0.057 | 0.145 |  |
| Age | 0.003 | 0.029 | 0.013 | 0.909 | 1.003 | [0.947, 1.063] |
| Education level | 0.338 | 0.146 | 5.390 | 0.020 | 1.403 | [1.054, 1.867] |
| SES residence | -0.082 | 0.051 | 2.521 | 0.112 | 0.922 | [0.833, 1.019] |
| Parity | 0.141 | 0.294 | 0.231 | 0.631 | 1.152 | [0.647, 2.051] |
| Activity | -0.369 | 0.321 | 1.322 | 0.250 | 0.691 | [0.368, 1.297] |
| Relationship status | 0.103 | 0.420 | 0.060 | 0.807 | 1.108 | [0.487, 2.522] |
| ACE total | 0.345 | 0.074 | 21.789 | <0.001 | 1.412 | [1.222, 1.632] |

Note: Nagelkerke R^2^ = 0.16, n= 243. Variables at time of recruitment. Coding: parity (1= 1 or more children, 0=no children), activity (1= Full/part-time work and have a job but not at work; 0 = all others), relationship status (1= de facto or married; 0 = all others).

**Table 2**. *Regression coefficients for predicting perinatal trauma, including the ACE abuse score.*

|  | B | SE B | Wald χ2 | p-value | OR | 95% CI OR |
| --- | --- | --- | --- | --- | --- | --- |
| Constant | -1.488 | 1.009 | 2.176 | 0.140 | 0.226 |  |
| Age | -0.009 | 0.031 | 0.082 | 0.775 | 0.991 | [0.933, 1.053] |
| Education level | 0.329 | 0.150 | 4.812 | 0.028 | 1.390 | [1.036, 1.866] |
| SES residence | -0.085 | 0.053 | 2.591 | 0.107 | 0.919 | [0.828, 1.019] |
| Parity | 0.139 | 0.303 | 0.211 | 0.646 | 1.149 | [0.634, 2.082] |
| Activity | -0.410 | 0.331 | 1.528 | 0.216 | 0.664 | [0.347, 1.271] |
| Relationship status | 0.223 | 0.442 | 0.254 | 0.615 | 1.249 | [0.525, 2.972] |
| ACE abuse | 1.133 | 0.214 | 28.014 | <0.001 | 3.104 | [2.041, 4.721] |

Note: Nagelkerke R^2^ = 0.22, n = 243.Variables at time of recruitment. Coding: parity (1= 1 or more children, 0=no children), activity (1= Full/part-time work and have a job but not at work; 0 = all others), relationship status (1= de facto or married; 0 = all others).

**Table 3**. *Regression coefficients for predicting perinatal trauma, including the ACE neglect score.*

|  | B | SE B | Wald χ2 | p-value | OR | 95% CI OR |
| --- | --- | --- | --- | --- | --- | --- |
| Constant | -1.140 | 0.983 | 1.344 | 0.246 | 0.320 |  |
| Age | 0.000 | 0.031 | 0.000 | 0.994 | 1.000 | [0.942, 1.061] |
| Education level | 0.336 | 0.150 | 5.028 | 0.025 | 1.399 | [1.043, 1.876] |
| SES residence | -0.096 | 0.052 | 3.438 | 0.064 | 0.909 | [0.822, 1.005] |
| Parity | -0.044 | 0.298 | 0.022 | 0.882 | 0.957 | [0.533, 1.716] |
| Activity | -0.323 | 0.323 | 0.999 | 0.318 | 0.724 | [0.385, 1.364] |
| Relationship status | -0.106 | 0.416 | 0.065 | 0.798 | 0.899 | [0.398, 2.031] |
| ACE neglect | 2.047 | 0.445 | 21.187 | <0.001 | 7.746 | [3.240, 18.522] |

Note: Nagelkerke R^2^ = 0.19, n = 243. Variables at time of recruitment. Coding: parity (1= 1 or more children, 0=no children), activity (1= Full/part-time work and have a job but not at work; 0 = all others), relationship status (1= de facto or married; 0 = all others).

**Table 4**. *Regression coefficients for predicting perinatal trauma, including the ACE dysfunction score.*

|  | B | SE B | Wald χ2 | p-value | OR | 95% CI OR |
| --- | --- | --- | --- | --- | --- | --- |
| Constant | -1.210 | 0.982 | 1.517 | 0.218 | 0.298 |  |
| Age | 0.003 | 0.028 | 0.015 | 0.903 | 1.003 | [0.950, 1.060] |
| Education level | 0.268 | 0.139 | 3.684 | 0.055 | 1.307 | [0.994, 1.717] |
| SES residence | -0.075 | 0.049 | 2.365 | 0.124 | 0.927 | [0.842, 1.021] |
| Parity | 0.126 | 0.283 | 0.198 | 0.656 | 1.134 | [0.652, 1.974] |
| Activity | -0.202 | 0.304 | 0.442 | 0.506 | 0.817 | [0.451, 1.481] |
| Relationship status | -0.068 | 0.391 | 0.030 | 0.863 | 0.935 | [0.435, 2.009] |
| ACE dysfunction | 0.273 | 0.109 | 6.251 | 0.012 | 1.314 | [1.061, 1.629] |

Note= Nagelkerke R^2^ = 0.06, n = 243. Variables at time of recruitment. Coding: parity (1= 1 or more children, 0=no children), activity (1= Full/part-time work and have a job but not at work; 0 = all others), relationship status (1= de facto or married; 0 = all others).

**Table 5**. *Regression coefficients for predicting perinatal trauma, including a total ACE of 4 or over vs 0*.

|  | B | SE B | Wald χ2 | p-value | OR | 95% CI OR |
| --- | --- | --- | --- | --- | --- | --- |
| Constant | -2.418 | 1.354 | 3.189 | 0.074 | 0.089 |  |
| Age | 0.005 | 0.037 | 0.017 | 0.896 | 1.005 | [0.934, 1.082] |
| Education level | 0.499 | 0.198 | 6.311 | 0.012 | 1.647 | [1.116, 2.430] |
| SES residence | -0.100 | 0.068 | 2.134 | 0.144 | 0.905 | [0.792, 1.035] |
| Parity | 0.246 | 0.398 | 0.383 | 0.536 | 1.279 | [0.586, 2.792] |
| Activity | -0.487 | 0.422 | 1.332 | 0.248 | 0.614 | [0.269, 1.405] |
| Relationship status | 0.162 | 0.562 | 0.083 | 0.773 | 1.175 | [0.391, 3.534] |
| ACE ≥4 vs 0 | 1.809 | 0.451 | 16.080 | <0.001 | 6.107 | [2.522, 14.788] |

Note= Nagelkerke R^2^ =. 0.20, n = 147. Variables at time of recruitment. Coding: parity (1= 1 or more children, 0=no children), activity (1= Full/part-time work and have a job but not at work; 0 = all others), relationship status (1= de facto or married; 0 = all others).

**Table 6**. *Regression coefficients for predicting perinatal trauma, including a total ACE of 4 or over vs less than 4.*

|  | B | SE B | Wald χ2 | p-value | OR | 95% CI OR |
| --- | --- | --- | --- | --- | --- | --- |
| Constant | -1.748 | 1.005 | 3.023 | 0.082 | 0.174 |  |
| Age | 0.002 | 0.029 | 0.003 | 0.953 | 1.002 | [0.946, 1.061] |
| Education level | 0.384 | 0.149 | 6.625 | 0.010 | 1.467 | [1.096, 1.965] |
| SES residence | -0.081 | 0.051 | 2.502 | 0.114 | 0.922 | [0.834, 1.020] |
| Parity | 0.027 | 0.293 | 0.008 | 0.928 | 1.027 | [0.578, 1.824] |
| Activity | -0.344 | 0.321 | 1.151 | 0.283 | 0.709 | [0.378, 1.329] |
| Relationship status | 0.054 | 0.413 | 0.017 | 0.895 | 1.056 | [0.470, 2.372] |
| ACE ≥4 vs <4 | 1.724 | 0.377 | 20.937 | <0.001 | 5.609 | [2.680, 11.738] |

Note= Nagelkerke R^2^ =. 0.15, n = 243. Variables at time of recruitment. Coding: parity (1= 1 or more children, 0=no children), activity (1= Full/part-time work and have a job but not at work; 0 = all others), relationship status (1= de facto or married; 0 = all others).

**Table 7.** *Regression coefficients for predicting PTSD severity, including having at least one ACE related to abuse*

|  | B | SE B | 95% CI | β | t | p-value |
| --- | --- | --- | --- | --- | --- | --- |
| Constant | 31.154 | 10.257 | [10.766, 51.542] |  | 3.037 | 0.003 |
| Age | 0.067 | 0.308 | [0-.546, 0.680] | 0.021 | 0.217 | 0.829 |
| Education level | -0.456 | 1.414 | [-3.266, 2.355] | -0.030 | -0.322 | 0.748 |
| SES residence | -0.993 | 0.498 | [-1.983, -0.003] | -0.179 | -1.995 | 0.049 |
| Parity | -7.879 | 2.924 | [-13.691, -2.066] | -0.248 | -2.694 | 0.008 |
| Activity | 7.866 | 3.104 | [1.696, 14.036] | 0.242 | 2.534 | 0.013 |
| Relationship status | -12.045 | 3.767 | [-19.533, -4.556] | -0.298 | -3.197 | 0.002 |
| ACE abuse ≥1 | 5.546 | 2.773 | [0.035, 11.057] | 0.183 | 2.000 | 0.049 |

Note= Nagelkerke R^2^= 0 29, n = 95, p <0.001. Variables at time of recruitment. Coding: parity (1= 1 or more children, 0=no children), activity (1= Full/part-time work and have a job but not at work; 0 = all others), relationship status (1= de facto or married; 0 = all others).

**Table 8**. *Regression coefficients for predicting PTSD severity, including having at least one ACE related to neglect*

|  | B | SE B | 95% CI | β | t | p-value |
| --- | --- | --- | --- | --- | --- | --- |
| Constant | 33.361 | 10.506 | [12.479, 54.243] |  | 3.175 | 0.002 |
| Age | 0.076 | 0.316 | [0-.553, 0.705] | 0.024 | 0.240 | 0.811 |
| Education level | -0.314 | 1.446 | [-3.188, 2.559] | -0.021 | -0.217 | 0.829 |
| SES residence | -1.041 | 0.509 | [-2.053, -.030] | -0.187 | -2.046 | 0.044 |
| Parity | -8.747 | 2.971 | [-14.652, -2.842] | -0.276 | -2.944 | 0.004 |
| Activity | 8.804 | 3.116 | [2.610, 14.998] | 0.271 | 2.825 | 0.006 |
| Relationship status | -12.643 | 3.825 | [-20.246, -5.040] | -0.313 | -3.305 | 0.001 |
| ACE neglect ≥1 | 2.743 | 3.034 | [-3.286, 8.773] | 0.084 | 0.904 | 0.368 |

Note= Nagelkerke R^2^= 0.27, n = 95, p<0.001. Variables at time of recruitment. Coding: parity (1= 1 or more children, 0=no children), activity (1= Full/part-time work and have a job but not at work; 0 = all others), relationship status (1= de facto or married; 0 = all others).

**Table 9**. *Regression coefficients for predicting PTSD severity, including having at least one ACE related to dysfunction*

|  | B | SE B | 95% CI | β | t | p-value |
| --- | --- | --- | --- | --- | --- | --- |
| Constant | 33.751 | 10.676 | [12.531, 54.972] |  | 3.161 | 0.002 |
| Age | 0.051 | 0.315 | [0-.575, 0.677] | 0.016 | 0.162 | 0.871 |
| Education level | -0.386 | 1.445 | [-3.259, 2.486] | -0.026 | -0.267 | 0.790 |
| SES residence | -0.970 | 0.510 | [-1.984, 0.045] | -0.174 | -1.900 | 0.061 |
| Parity | -8.419 | 2.970 | [-14.322,-2.515] | -0.265 | -2.834 | 0.006 |
| Activity | 8.809 | 3.153 | [2.542, 15.076] | 0.271 | 2.794 | 0.006 |
| Relationship status | -12.970 | 3.813 | [-20.550,-5.391] | -0.321 | -3.401 | 0.001 |
| ACE dysfunction ≥1 | 1.806 | 2.900 | [-3.957, 7.570] | 0.057 | 0.623 | 0.535 |

Note= Nagelkerke R^2^= 0.26, n = 95, p<0.001. Variables at time of recruitment. Coding: parity (1= 1 or more children, 0=no children), activity (1= Full/part-time work and have a job but not at work; 0 = all others), relationship status (1= de facto or married; 0 = all others).
